# Supplementary material for: Polygenic hazard score is associated with prostate cancer in multi-ethnic populations
Source: Nat Commun. 2021 Feb 23;12:1236. doi: 10.1038/s41467-021-21287-0 (PMC7902617; doi:10.1038/s41467-021-21287-0)
Supplement: Supplementary file 1 — Supplementary Information [file 41467_2021_21287_MOESM1_ESM.pdf]

Supplementary Information

Supplementary Table 1. Study names, details, and participant information obtained from the OncoArray project[1,2].

**Study Information**				**Participants**				**OncoArray Genetic Ancestry***						
**Abbreviation**	**Name**	**Location**	**Design**	**Controls**	**Any PCa**	**Aggressive PCa**	**Died of PCa**	**European**	**Asian**	**African**	**Known PCa Family History**	**PHS_2 (median, IQR)**	**Age at PCa Diagnosis (median, IQR)**	**Age at Last Follow-up (median, IQR)**
Aarhus (n=1630)	Aarhus Prostate Cancer Study	Aarhus, Denmark	Hospital-based, observational	550	1080	839	65	1621	9	0	856	0.4892 [0.2875, 0.7081]	64.1 [59.8, 68.0]	67.1 [62.3, 71.9]
AHS (n=1650)	Agricultural Health Study	Maryland, USA	Case-control (nested) within prospective cohort	1159	491	238	23	1650	0	0%	1572	0.4193 [0.2232, 0.6236]	67.6 [61.8, 73.5]	75.0 [69.0, 81.2]
ATBC (n=3117)	Alpha-Tocopherol Beta-Carotene	Maryland, USA	Prospective, nested case-control	1910	1207	252	238	3117	0	0	2761	0.4649 [0.2686, 0.6628]	72.0 [69.0, 76.0]	78.6 [74.0, 82.6]
BioVU (n=204)	Vanderbilt University Biorepository	Nashville, USA	Hospital-based, case-control	0	204	0	0	0	0	204	0	0.4768 [0.2658, 0.6174]	61.0 [55.4, 66.5]	61.0 [55.4, 66.5]
Canary PASS (n=373)	Prostate Active Surveillance Study	USA	Prospective, observational	0	373	0	0	364	9	0	0	0.5186 [0.3357, 0.7402]	63.0 [58.0, 67.0]	63.0 [58.0, 67.0]
CCI (n=270)	CCI Prostate	Alberta, Canada	Hospital-based, case series	0	270	71	1	266	4	0	0	0.5666 [0.381, 0.7521]	64.3 [59.1, 68.7]	69.8 [64.7, 75.5]
CeRePP (n=1755)	French Prostate Case Control Study	Paris, France	Case-control, prospective, hospital-based	730	1025	805	13	1567	3	185	1748	0.4935 [0.2943, 0.6984]	66.0 [60.0, 71.0]	68.0 [61.0, 73.0]
CHIPGECS (n=1070)	Chinese Prostate Cancer Genetic and Environmental Correlation Study	China	Population-based, case-control, observational	596	474	293	0	0	1070	0	0	0.3456 [0.1343, 0.5244]	71.0 [65.0, 76.0]	70.0 [63.0, 75.0]
COH (n=519)	City of Hope	Duarte, USA	Case-control	259	260	192	0	516	3	0	260	0.496 [0.2931, 0.7019]	62.0 [55.0, 65.0]	61.0 [56.0, 66.1]
COSM (n=3282)	Cohort of Swedish Men	Stockholm, Sweden	Population-based cohort	1117	2165	1404	278	3279	3	0	2455	0.4861 [0.2975, 0.6946]	69.9 [64.6, 76.5]	78.0 [72.3, 84.1]

CPCS1 (n=791)	Copenhagen Prostate Cancer Study 1	Copenhagen, Denmark	Case-control	256	535	433	8	790	1	0	0	0.4833 [0.2922, 0.692]	68.1 [63.9, 72.6]	70.4 [64.6, 76.2]
CPCS2 (n=667)	Copenhagen Prostate Cancer Study 2	Copenhagen, Denmark	Case-control	227	440	323	3	667	0	0	0	0.483 [0.284, 0.6848]	64.4 [60.7, 67.7]	67.8 [61.0, 71.7]
CPDR (n=176)	Center for Prostate Disease Research	USA	Retrospective	41	135	38	0	0	0	176	153	0.4088 [0.2264, 0.5656]	56.0 [50.7, 62.1]	57.7 [52.7, 65.0]
EPIC (n=1328)	European Prospective Investigation Into Cancer and Nutrition (BPC3)	European Union	Case-control	696	632	180	28	1324	4	0	0	0.4668 [0.2676, 0.6722]	67.2 [62.7, 71.0]	70.3 [65.2, 73.7]
EPICAP (n=29)	EPIdemiologcal study of Prostate CAncer	Hérault, France	Population-based, case-control	9	20	15	3	0	0	29	18	0.47315 [0.2626, 0.583]	64.6 [61.8, 68.6]	64.9 [61.5, 68.5]
ERSPC (n=137)	Erasmus Medical Centre	Rotterdam, The Netherlands	Population-based, randomized control trial	66	71	0	0	136	1	0	0	0.4597 [0.2111, 0.6343]	71.0 [69.5, 73.2]	71.1 [69.5, 73.1]
ESTHER (n=17)	Epidemiological investigations of the chances of preventing, recognizing early and optimally treating chronic diseases in an elderly population	Heidelberg, Germany	Population-based, prospective, case-control	3	14	6	0	17	0	0	17	0.4385 [0.183, 0.5686]	66.6 [64.8, 67.8]	70.8 [67.8, 73.1]
FHCRC (n=812)	Fred Hutchinson Cancer Research Centre	Seattle, USA	Population-based, case control	399	413	226	28	795	17	0	812	0.4739 [0.2901, 0.6608]	60. [55.0, 66.0]	68.0 [60.0, 75.4]
Gene-PARE (n=253)	Genetic Predictors of Adverse Radiotherapy Effects	New York, USA and Tokyo/Chiba, Japan	Hospital-based	0	253	125	0	242	11	0	0	0.56415 [0.3733, 0.73184]	66.3 [61.3, 71.8]	75.3 [69.1, 79.8]
Hamburg-Zagreb (n=295)	Hamburg-Zagreb	Hamburg, Germany	Hospital-based, prospective	149	146	112	10	295	0	0	267	0.4992 [0.2738, 0.6679]	68.3 [62.3, 74.0]	62.0 [54.0, 73.4]

HPFS (n=2221)	Health Professionals Follow-up Study	Boston, USA	Nested case-control	1050	1171	581	75	2212	9	0	368	0.4676 [0.2646, 0.6534]	70.0 [65.0, 75.0]	79.7 [73.7, 84.7]
IMPACT (n=925)	Identification of Men with a genetic predisposition to ProstAte Cancer	The ICR, London, UK	Observational	875	50	24	0	915	10	0	925	0.433 [0.2271, 0.6234]	64.3 [61.0, 67.3]	57.3 [49.9, 64.7]
IPO-Porto (n=551)	Portuguese Oncology Institute	Porto, Portugal	Hospital-based	180	371	276	1	551	0	0	371	0.547 [0.3052, 0.7734]	55.0 [52.0, 61.0]	60.0 [54.0, 65.4]
KARUPROSTATE (n=749)	French West Indies Prostate Cancer Study	French West Indies	Case-control	386	363	226	0	0	0	749	735	0.3629 [0.2113, 0.5466]	66.7 [61.0, 72.0]	63.5 [57.3, 70.2]
KULEUVEN (n=269)	Katholieke Universiteit Leuven	Leuven, Belgium	Hospital-based, prospective, observational	103	166	149	8	269	0	0	40	0.4419 [0.2169, 0.6643]	66.9 [60.4, 71.0]	74.3 [68.5, 80.2]
LAAPC (n=732)	Los Angeles Study of Aggressive Prostate Cancer	California, USA	Population-based, case control	285	447	0	0	720	12	0	0	0.4691 [0.274, 0.6769]	67.0 [60.0, 74.0]	66.0 [58.0, 73.0]
Malaysia (n=405)	Malaysia	Malaysia	Case-control	202	203	175	18	1	404	0	392	0.4113 [0.2112, 0.6088]	70.5 [65.4, 75.5]	75.2 [71.1, 79.5]
MCC-Spain (n=922)	Multi Case-Control Study-Spain	Barcelona, Spain	Case-control	399	523	356	7	917	5	0	879	0.5136 [0.3101, 0.7001]	66.7 [61.7, 72.2]	70.3 [64.5, 76.0]
MCCS (n=1033)	Melbourne Collaborative Cohort Study	Melbourne, Australia	Nested case-control	315	718	374	81	1030	3	0	134	0.5062 [0.2991, 0.7209]	69.2 [65.0, 74.4]	79.2 [73.9, 84.0]
MDACC_AS (n=535)	MD Anderson Cancer Center Active Surveillance Trial	Texas, USA	Prospective, cohort	0	535	151	0	501	34	0	534	0.5305 [0.3522, 0.7361]	64.9 [58.6, 70.3]	64.9 [58.6, 70.3]
MEC (n=1298)	Multiethnic Cohort Study	California / Hawaii, USA	Population-based	670	628	0	29	1240	58	0	1231	0.4677 [0.2608, 0.6595]	69.8 [64.2, 75.3]	79.5 [73.4, 84.7]
MIAMI-WFPCS (n=108)	Miami-Wake Forest Prostate Cancer Study	North Carolina, USA	Case-control	49	59	25	0	0	0	108	107	0.3724 [0.2112, 0.5631]	60.4 [54.8, 65.0]	58.1 [53.0, 64.4]
MOFFITT (n=797)	Moffitt Prostate Cancer Study	Florida, USA	Hospital-based	296	501	268	4	596	9	192	794	0.4863 [0.2998, 0.677]	64.0 [58.7, 69.4]	64.1 [57.3, 71.3]
NMHS (n=364)	Nashville Men's Health Study	Nashville, USA	Clinic-based, case-control	188	176	95	0	0	0	364	364	0.4022 [0.2099, 0.5637]	64.0 [57.0, 69.0]	63.0 [57.0, 68.0]

Oslo (n=1453)	Oslo University Hospital / General Cohort of Adults in Norway (CONOR)	Oslo, Norway	Population-based, observational	0	1453	51	764	1443	10	0	0	0.5692 [0.3692, 0.7409]	72.9 [66.5, 78.4]	79.2 [73.0, 84.3]
PCaP (n=967)	North Carolina-Louisiana Prostate Cancer Project	North Carolina / Louisiana, USA	Population-based cohort	0	967	432	0	0	0	967	967	0.4407 [0.2784, 0.601]	62.0 [56.0, 68.0]	62.0 [56.0, 68.0]
PCMUS (n=281)	Prostate Cancer Study Medical University Sofia	Sofia, Bulgaria	Case-control	89	192	171	0	281	0	0	252	0.4976 [0.3215, 0.7323]	68.0 [63.0, 74.0]	67.9 [62.0, 74.0]
PHS (n=898)	Physicians Health Study	Harvard, Boston, USA	Nested case-control	267	631	375	115	878	20	0	70	0.4947 [0.2983, 0.7133]	68.5 [63.5, 73.5]	82.7 [77.8, 87.3]
PLCO (n=1657)	Prostate, Lung, Colorectal, and Ovarian Cancer Screening Trial	Bethesda, USA	Nested case-control	980	677	389	8	1657	0	0	1629	0.4371 [0.253, 0.6419]	72.0 [68.0, 76.0]	74.0 [70.0, 78.0]
Poland (n=801)	The Poland Group	Szczecin, Poland	Case-control	317	484	373	0	800	1	0	437	0.5143 [0.3311, 0.7036]	69.0 [63.0, 76.0]	67.9 [61.0, 74.9]
PRAGGA (n=232)	Prostate cAncer Genetics in Galicia	Galicia, Spain	Case-control	102	130	88	6	229	3	0	204	0.4896 [0.3285, 0.7238]	68.9 [64.6, 72.3]	73.2 [62.8, 78.8]
PROCAP (n=868)	PROgression in Cancer of the Prostate	Stockholm, Sweden	Population-based, observational	241	627	310	215	862	6	0	0	0.5326 [0.317, 0.739]	65.3 [61.3, 68.5]	74.9 [69.2, 79.2]
PROFILE (n=35)	Genetic prostate cancer risk stratification for targeted screening	The ICR, London, UK	Hospital-based, observational, prospective	22	13	7	0	34	1	0	35	0.4871 [0.2868, 0.6777]	61.1 [53.7, 66.8]	57.1 [48.6, 63.0]
PROGReSS (n=996)	Prostate cancer: Mechanisms of Progression and Treatment	Santiago de Compostela, Spain	Hospital-based, observational, prospective	322	674	465	21	995	1	0	174	0.5012 [0.3041, 0.6955]	71.0 [64.7, 75.1]	74.2 [65.5, 79.2]
ProMPT (n=798)	Prostate Cancer: Mechanisms of Progression and Treatment	Cambridge, UK	Population-based	12	786	627	37	791	7	0	156	0.5924 [0.4041, 0.809]	64.5 [60.0, 69.2]	68.2 [63.6, 73.6]

ProtecT (n=1419)	Prostate Testing for Cancer and Treatment	Oxford, Bristol, and Cambridge, UK	Population-based cohort	1415	4	0	0	1413	6	0	1242	0.4101 [0.1987, 0.5984]	64.1 [60.7, 67.9]	61.4 [57.2, 65.4]
PROtEuS (n=123)	The Prostate Cancer and Environment Study	Montreal, Canada	Population-based, case-control	53	70	45	0	0	0	123	118	0.4057 [0.2222, 0.572]	63.5 [57.2, 66.5]	63.9 [59.4, 68.6]
QLD (n=4564)	Queensland Study	Brisbane, Australia	Case-control	1252	3312	2680	53	4504	60	0	2165	0.526 [0.3248, 0.7249]	62.7 [57.8, 67.2]	66.1 [60.4, 71.5]
RAPPER (n=2117)	Radiogenomics: Assessment of Polymorphisms for Predicting the Effects of Radiotherapy	Manchester, UK	Hospital-based, prospective	0	2117	0	0	2100	17	0	0	0.573 [0.3838, 0.7748]	70.6 [66.0, 74.7]	70.6 [66.0, 74.7]
SABOR (n=211)	San Antonio Center of Biomarkers of Risk for Prostate Cancer	San Antonio, USA	Population-based cohort	106	105	37	0	0	0	211	0	0.4537 [0.2669, 0.6288]	63.3 [55.8, 70.2]	63.6 [55.9, 70.4]
SCCS (n=1789)	Southern Community Cohort Study	Southeastern USA	Population-based cohort	1498	291	20	0	0	0	1789	1665	0.3858 [0.2208, 0.5602]	58.0 [53.0, 64.0]	60.0 [53.0, 67.0]
SCPCS (n=89)	South Carolina Prostate Cancer Study	South Carolina, USA	Population-based, case control, observational	32	57	26	0	0	0	89	89	0.4141 [0.2064, 0.5879]	71.0 [68.0, 75.0]	70.0 [67.0, 74.0]
SEARCH (n=2669)	Study of Epidemiology and Risk factors in Cancer Heredity	Cambridge, UK	Case-control	232	2437	1549	148	2637	32	0	1519	0.5569 [0.3672, 0.76]	64.0 [61.0, 67.0]	70.8 [66.6, 74.0]
SFPCS (n=602)	San Francisco Prostate Cancer Study	California, USA	Population-based, case control, observational	241	361	55	50	484	1	117	602	0.4776 [0.2926, 0.6781]	65.0 [58.6, 71.0]	69.1 [62.6, 76.0]
SNP_Prostate_Ghent (n=451)	SNP Prostate Ghent	Ghent, Belgium	Hospital-based, observational	135	316	243	1	451	0	0	393	0.5033 [0.2725, 0.7168]	65.4 [60.6, 70.3]	68.8 [62.8, 75.0]

SPAG (n=211)	Serum Proteomic analysis for biomarkers of Aggressive prostate disease in the Guernsey population	Manchester / Southampton, UK	Hospital-based, observational	172	39	30	1	210	1	0	10	0.4637 [0.2589, 0.658]	66.1 [62.1, 68.5]	68.6 [62.8, 74.4]
STHM2 (n=4524)	Stockholm 2	Stockholm, Sweden	Population-based, observational	1491	3033	1562	96	4497	27	0	0	0.5051 [0.3035, 0.6941]	65.6 [60.9, 70.1]	69.7 [64.6, 74.5]
SWOG-PCPT (n=2409)	Prostate Cancer Prevention Trial	Seattle, USA	Case-control from randomized trial	1273	1136	338	0	2154	91	164	2409	0.4246 [0.2374, 0.6161]	69.0 [65.0, 74.0]	74.4 [70.0, 79.8]
SWOG-SELECT (n=3849)	Selenium and Vitamin E Cancer Prevention Trial	Seattle, USA	Case-control from randomized trial	2307	1542	561	10	3541	111	197	3848	0.4448 [0.2441, 0.6349]	67.0 [63.0, 71.0]	67.0 [62.0, 73.0]
TAMPERE (n=3591)	Finnish Genetic Predisposition to Prostate Cancer Study	Tampere, Finland	Population-based, case-control, observational	1179	2412	1478	146	3585	6	0	134	0.5107 [0.3098, 0.7065]	67.1 [62.8, 71.3]	73.6 [69.5, 78.0]
TORONTO (n=1276)	Toronto	Toronto, Canada	Prospective, hospital-based cohort	544	732	411	0	1123	153	0	1166	0.4956 [0.2958, 0.6852]	65.0 [60.2, 70.6]	63.8 [58.3, 69.3]
UKGPCS (n=11021)	U.K. Genetic Prostate Cancer Study and The Prostate Cancer Research Foundation Study	United Kingdom	Hospital-based	2973	8048	5107	1378	10514	142	365	7772	0.5358 [0.3319, 0.7445]	58.7 [55.2, 63.6]	66.0 [61.7, 73.0]
ULM (n=408)	Institut fuer Humangengetik Ulm	Ulm, Germany	Case-control	3	405	307	13	407	1	0	408	0.5654 [0.3859, 0.7537]	65.0 [60.0, 70.3]	68.6 [63.1, 74.0]
WUGS (n=898)	Washington University Genetics Study	St. Louis, USA	Case series, hospital-based	152	746	430	0	668	6	224	773	0.5091 [0.3173, 0.7336]	62.0 [56.0, 67.0]	64.0 [57.0, 69.0]
**Overall (n=80491)**				**30575**	**49916**	**26419**	**3983**	**71856**	**2382**	**6253**	**46030**	**0.4908 [0.2910, 0.6908]**	**65.3 [59.6, 71.0]**	**70.0 [63.4, 76.5]**

* Genetic ancestry definitions shown here were previously defined using a principal components analysis in OncoArray.

Explorations of Genetic Ancestry

A comparison of self-reported race/ethnicity and OncoArray-defined genetic ancestry[2,3] is shown in Supplementary Table 2. The performance of PHS was also evaluated using participants' self-reported race/ethnicity. Based on prior power analyses, subgroups with ≥1,000 men were analyzed[4]: European; Black or African American; and East Asian. Those with other/unknown self-reported race/ethnicity were excluded. Model performance was assessed with $HR_{80/20}$.

Supplementary Table 2. Self-reported race/ethnicity and OncoArray-defined genetic ancestry for individuals in the dataset (n=80,491).

Self-reported Race/Ethnicity	Number	OncoArray Genetic Ancestry		
		European	Asian	African
European	63,954	63,685	250	19
Black or African American*	6,247	29	16	6,202
East Asian	1,212	21	1,190	1
Hawaiian	154	138	16	0
Hispanic American	326	139	176	11
South Asian	167	17	150	0
Other/Unknown	8,431	7,827	584	20

*Includes African American, Black African, Black Caribbean

We also evaluated genetic ancestry and admixture using fastSTRUCTURE[5], generating agnostic clusters of the data into *K*=2-6 populations. Supplementary Table 3 shows a comparison of fastSTRUCTURE clustering, OncoArray-defined genetic ancestry, and self-reported race/ethnicity.

Supplementary Table 3. Comparison of fastSTRUCTURE genetic clustering against OncoArray-defined genetic ancestry, and self-reported race/ethnicity for individuals in the dataset (n=80,491).

		**OncoArray-defined Genotypic Ancestry**				**Self-reported Race/Ethnicity**						
		European	Asian	African		European	East Asian	Black or African American	Hawaiian	Hispanic American	South Asian	Other/ Unknown
**fastSTRUCTURE *K***	**Cluster**											
K=2	1	71572	44	0		63514	19	27	136	110	17	7793
	2	0	0	4515		1	0	4507	0	0	0	7
	Admixed	284	2338	1738		439	1193	1713	18	216	150	631
K=3	1	19959	238	3		17923	6	10	44	91	54	2072
	2	0	2	5483		1	0	5472	0	2	0	10
	3	42128	96	7		37056	9	21	72	89	21	4963
	Admixed	9769	2046	760		8974	1197	744	38	144	92	1386
K=4	1	0	0	5043		1	0	5034	0	0	0	8
	2	8803	958	21		7971	488	21	21	127	61	1093
	3	24801	160	8		22043	9	20	50	74	45	2728
	4	26459	84	7		23242	8	17	57	57	25	3144
	Admixed	11793	1180	1174		10697	707	1155	26	68	36	1458
K=5	1	22760	79	2		20214	36	12	43	28	7	2501
	2	695	47	4346		622	20	4337	2	3	6	98
	3	25057	435	13		22251	227	27	48	91	24	2837
	4	4223	400	1397		3771	194	1386	15	29	27	598
	5	8899	757	144		7910	418	143	23	85	46	1175
	Admixed	10222	664	351		9186	317	342	23	90	57	1222

K=6	1	428	284	5095		406	199	5087	4	2	4	105
	2	3401	305	42		3193	58	47	8	76	30	336
	3	7724	258	372		6776	38	364	25	97	66	988
	4	12466	70	255		11170	20	255	19	28	10	1289
	5	16748	9	9		14658	3	12	35	14	3	2041
	6	14789	81	64		13130	8	71	36	36	13	1640
	Admixed	16300	1375	416		14621	886	411	27	73	41	2032

Supplementary Methods

Adapting the PHS to OncoArray: Proxy SNP Identification

To identify proxy single-nucleotide polymorphisms (SNPs) for those not directly genotyped on OncoArray, we identified samples from 12,107 men of European genetic ancestry that were genotyped on both the iCOGs array and OncoArray, within the PRACTICAL consortium. We searched for proxy SNPs (on the same chromosome as the original SNP and within 10^6 base pairs of the original SNP) with linkage disequilibrium $r^2 \geq 0.90$. Since a suitable proxy could not be identified on OncoArray for all 30 missing SNPs, we re-calculated the β parameter estimates for a Cox proportional hazards regression, using the same dataset used for the initial development of PHS_1. That development dataset includes 18,868 prostate cancer cases and 12,879 controls and has been described previously[6].

PHS percentile thresholds

Percentiles of PHS_2 were determined from the original reference dataset for PHS_1 (i.e., controls in the development set with age <70 years[6]). Percentiles for PHS_2 are shown in Supplementary Table 4.

Supplementary Table 4. Percentile thresholds of PHS_2.

**Percentile**	**PHS_2**
20th	0.1603
50th	0.3949
80th	0.6521
98th	0.9966

Sample-Weight Correction and Sensitivity Analysis

In order to appropriately calculate the sample-weight corrections, we needed prostate cancer age-specific incidence rates at a population level. We were able to obtain such information for the following countries: the USA[7], Sweden[8–11], Norway[8,9], Finland[8,9], Denmark[8,9], the UK[12], and Australia[13]. While the incidence rates in each age range varied between countries (likely a reflection of screening prevalence and differing policies for prostate cancer screening in each population), the pattern was relatively similar, and typically varied by a factor of less than 2-3 for each 5-year age bin. Sweden was chosen as a representative population with published raw numbers of men diagnosed with prostate cancer[10,11] and of total men in the age range of 45-79 years. Published Swedish Cancer Registry and Swedish population data reported there were 9,024 prostate cancer cases and 1,953,203 men without prostate cancer aged 45-79 in 2016[10,11]. We used these numbers to correct for sampling bias[14,15] for all Cox proportional hazards regressions reported; calculations were made using the R "survival" package[16].

Given that the overall incidence of prostate cancer in different populations varies, and the current dataset comes from a multi-institutional and multi-national dataset, we performed a sensitivity analysis for the above population numbers. This was done by adjusting the assumed population prostate cancer incidence by a factor of up to four times lesser or greater than that of Sweden, then re-calculating the corrected hazard ratios (HRs) for PHS associations with PCa. The range of incidence in the sensitivity analyses (25% to 400% of the Swedish incidence) is wider than the range across the countries mentioned above and wider than the range reported for ethnicity-related variation[17,18]. We calculated each HR of interest ($HR_{20/50}$, $HR_{80/50}$, $HR_{98/50}$, and $HR_{80/20}$), for the association of PHS with any PCa, aggressive PCa, and death from PCa (in the multi-ethnic dataset and by each genetic ancestry group, where applicable) to determine how changes in assumed population incidence affected the calculated HRs.

SNPs included in PHS_2

Supplementary Table 5. SNP identifier, chromosome, effect allele, reference allele, and position (based on version 37) and beta (model weight) for the 46 SNPs used in PHS_2 calculation*.

ID	Chromosome	Effect	Reference	Position	PHS_2 beta
rs6545977	2	A	G	63301164	-0.069
rs1010 †	2	G	A	85808982	-0.049
rs16860513	2	T	A	173342367	-0.198
rs75219487	3	T	A	87147922	0.123
rs6788616	3	G	A	87205079	0.041
rs4857841 †	3	A	G	128046643	0.031
rs78416326	3	G	C	170074517	-0.080
rs6853490	4	G	A	95544718	0.053
rs17021918 §	4	A	G	95562877	-0.026
rs7679673 §	4	A	C	106061534	-0.071
rs7725218	5	A	G	1282414	-0.078
rs2736108	5	A	G	1297488	0.056
rs10866527	5	A	G	1891800	0.045
rs3910736	6	A	G	153412476	-0.073
rs7769879	6	C	G	160865645	0.053
rs6965016 †	7	C	A	97807882	0.049
rs11782388 †	8	G	A	23525358	0.062
rs9297746	8	G	A	127909361	-0.061
rs28556804	8	G	A	128014315	-0.074
rs77541621	8	A	G	128077146	0.200
rs1016343	8	A	G	128093297	0.073
rs60163266	8	A	G	128323157	0.073
rs6983267 §	8	A	C	128413305	-0.100
rs7812894 †	8	T	A	128520479	0.084
rs12549761	8	G	C	128540776	-0.065
rs10993994 §	10	A	G	51549496	0.106
rs72853963	11	A	G	2224664	0.071
rs12275055	11	G	A	68981359	0.083
rs7929962 †	11	G	A	68985583	-0.047
rs11568818 §	11	G	A	102401661	-0.047
rs10875943 §	12	G	A	49676010	0.036
rs55914512	12	A	C	53282274	0.050
rs4643253	14	G	A	69106108	-0.049
rs684232 §	17	G	A	618965	0.042
rs718961	17	A	G	36077099	-0.074
rs11651052 †	17	A	G	36102381	-0.094
rs117576373	17	A	G	46820676	0.138
rs9889335 †	17	A	C	69115146	0.082
rs11672691 §	19	A	G	41985587	-0.062
rs17632542	19	G	A	51361757	-0.160

rs35897249	20	G	A	62233638	-0.049
rs73179053	22	G	A	43501620	-0.081
rs747745	22	G	A	43503547	-0.047
rs4907775 †	23	G	A	51263200	0.056
rs7888856	23	G	A	66751555	-0.047
rs11795627	23	A	G	69957441	-0.040

* Comparing the 46 SNPs included in PHS_2 and the 147 SNPs identified in a recent meta-analysis of men with European ancestry[1], there were 8 PHS_2 SNPs that were exact matches (§) and 9 PHS_2 SNPs in linkage disequilibrium (> 0.90, among men with European ancestry in the present study) (†) with one of the 147 meta-analysis SNPs.

Supplementary Table 6: Summary statistics for PHS_2.

	OncoArray-defined Genetic Ancestry			
**PHS_2 Summary Statistics**	**All**	**European**	**Asian**	**African**
Median PHS_2 (IQR)	0.49 (0.29, 0.69)	0.50 (0.30, 0.70)	0.41 (0.21, 0.60)	0.41 (0.24, 0.57)
Median PHS_2 (IQR) in controls	0.39 (0.20, 0.58)	0.40 (0.21, 0.59)	0.34 (0.14, 0.52)	0.37 (0.20, 0.54)
Median PHS_2 (IQR) in participants diagnosed with any prostate cancer	0.55 (0.36, 0.75)	0.56 (0.37, 0.76)	0.47 (0.27, 0.68)	0.43 (0.27, 0.60)
Median PHS_2 (IQR) in participants diagnosed with aggressive prostate cancer	0.55 (0.36, 0.75)	0.56 (0.37, 0.76)	0.46 (0.25, 0.67)	0.42 (0.27, 0.59)
Median PHS_2 (IQR) in participants who died of prostate cancer	0.56 (0.37, 0.76)	0.56 (0.37, 0.75)	0.51 (0.28, 0.74)	0.44 (0.31, 0.53)
Median PHS_2 (IQR) in participants who did not die of prostate cancer	0.49 (0.29, 0.69)	0.50 (0.30, 0.70)	0.40 (0.21, 0.59)	0.41 (0.24, 0.57)

Sensitivity Analyses

Supplementary Table 7: Results of the sensitivity analysis for PHS association with prostate cancer in the multi-ethnic dataset, and by genetic ancestry group. Results are the maximum deviation in each hazard ratio (HR) when the assumed population incidence was varied over a range of values, from 25% to 400% of that reported in Sweden. Each HR was within 2.5% of those reported in the manuscript, even with a 4-fold change (lesser or greater) in the assumed population incidence.

	**Maximum deviation of HRs comparing percentiles of PHS_2**			
OncoArray Genetic ancestry	**$HR_{20/50}$: ≤20th vs 30-70th**	**$HR_{80/50}$: ≥80th vs 30-70th**	**$HR_{98/50}$: ≥98th vs 30-70th**	**$HR_{80/20}$: ≥80th vs ≤20th**
	**Any prostate cancer**			
**All (n=80,491)**	1.1%	1.2%	1.9%	2.2%
**European (n=71,856)**	1.0%	1.1%	1.8%	2.0%
**Asian (n=2,382)**	1.2%	1.3%	2.1%	2.5%
**African (n=6,253)**	1.2%	1.1%	2.0%	2.2%
	**Aggressive prostate cancer**			
**All (n=58,600)**	1.1%	1.2%	1.9%	2.2%
**European (n=53,608)**	1.0%	1.1%	1.7%	2.0%
**Asian (n=1,806)**	1.1%	1.2%	2.0%	2.3%
**African (n=3,186)**	1.1%	1.1%	1.9%	2.2%
	**Fatal prostate cancer**			
**All (n=78,221)**	1.8%	1.9%	3.2%	3.7%

PHS and Family History: Age at Diagnosis of Aggressive Prostate Cancer

Supplementary Table 8: Multivariable models with both PHS and family history of prostate cancer (≥1 first-degree relative affected) for association with aggressive and fatal prostate cancer in the multi-ethnic dataset. Only individuals with known family history were included in these models. Both family history and PHS were significantly associated with aggressive prostate cancer and prostate cancer death in the combined models. Hazard ratios (HR) for family history were calculated as the exponent of the beta from the multivariable Cox proportional hazards regression[19]. The HR for PHS in the multivariable models was estimated as the $HR_{80/20}$ (men in the highest 20% vs. those in the lowest 20% of genetic risk by PHS_2) in each cohort. P-values reported are two-tailed from the Cox models. The models with PHS for association with aggressive (log-likelihood $p<10^{-300}$) and fatal (log-likelihood $p=7.8x10^{-59}$) prostate cancer performed better than the models with family history alone.

OncoArray Genetic Ancestry	**Variable**	**beta**	***z*-score**	**p-value**	**HR**
	**Aggressive Prostate Cancer**				
**All (n=35,852)**	PHS	2.16	47.8	$<10^{-300}$	5.12
	Family History	0.93	31.4	2.0×10^{-216}	2.54
**European (n=32,019)**	PHS	2.10	46.7	$<10^{-300}$	4.93
	Family History	0.92	31.4	2.0×10^{-216}	2.52
**Asian (n=902)**	PHS	1.73	38.9	10^{-300}	3.60
	Family History	0.33	6.7	2.1×10^{-11}	1.39
**African (n=2,931)**	PHS	1.10	22.1	3.2×10^{-108}	2.16
	Family History	0.94	31.5	8.7×10^{-218}	2.57
	**Fatal Prostate Cancer**				
**All (n=46,030)**	PHS	2.20	16.3	9.9×10^{-60}	5.29
	Family History	0.78	8.5	1.9×10^{-17}	2.18

<u>Explorations of Alternative Ancestry Groupings</u>

Self-reported race/ethnicity

Supplementary Table 9: Association of PHS with aggressive prostate cancer, by self-reported race/ethnicity. Hazard ratios (HRs) are shown comparing men in highest 20% of genetic risk (≥80th percentile) vs. the lowest 20% of genetic risk (≤20th percentile).

Self-reported Race/Ethnicity	$HR_{80/20}$: ≥80th vs ≤20th
European	5.52 [5.48, 5.57]
Black or African American	2.40 [2.36, 2.44]
East Asian	2.85 [2.74, 2.98]

fastSTRUCTURE

Supplementary Table 10. Association of PHS with aggressive prostate cancer, by *K*=2-6 clusters using fastSTRUCTURE. Hazard ratios (HRs) are shown comparing men in highest 20% of genetic risk (≥80th percentile) vs. the lowest 20% of genetic risk (≤20th percentile).

fastSTRUCTURE *K*	Cluster	$HR_{80/20}$: ≥80th vs ≤20th
K=2	1	5.60 [5.55, 5.64]
	2	2.06 [2.03, 2.09]
	Admixed	5.05 [4.89, 5.21]
K=3	1	5.75 [5.66, 5.83]
	2	2.43 [2.39, 2.47]
	3	5.42 [5.37, 5.47]
	Admixed	6.37 [6.25, 6.50]
K=4	1	2.17 [2.13, 2.20]
	2	5.28 [5.19, 5.40]
	3	5.75 [5.68, 5.83]
	4	5.56 [5.49, 5.63]
	Admixed	6.07 [5.97, 6.19]
K=5	1	6.06 [5.97, 6.14]
	2	6.31 [6.09, 6.54]
	3	6.09 [6.01, 6.29]

	4	6.11 [5.94, 6.29]
	5	5.09 [5.00, 5.20]
	Admixed	4.85 [4.76, 4.94]
K=6	1	5.32 [5.14, 5.49]
	2	6.41 [6.19, 6.66]
	3	4.76 [4.67, 4.87]
	4	6.62 [6.50, 6.75]
	5	5.18 [5.10, 5.26]
	6	5.57 [5.47, 5.66]
	Admixed	5.91 [5.83, 6.01]

Additional PRACTICAL Consortium Collaborators

Artitaya Lophatananon[1]
Teuvo L. J. Tammela[2]
Csilla Sipeky[3]
Anssi Auvinen[4]
Alison M. Dunning[5]
Maya Ghoussaini[6]
Suzanne Chambers[7,8]
Judith A. Clements[9,10]
Lisa Horvath[11,12]
Leire Moya[13,10]
Gail P. Risbridger[14,15]
Wayne Tilley[16]
Markus Aly[17,18]
Tobias Nordström[17,19]
Stig E. Bojesen[20,21]
Peter Iversen[22]
Martin Andreas Røder[23]
Phyllis J. Goodman[24]
Ian M. Thompson Jr.[25]
Melissa C. Southey[26,27,28]
Robert J. MacInnis[27,29]
Roger L. Milne[30,31,32]
Niclas Håkansson[33]
Stephanie Weinstein[34]
Fredrick R. Schumacher[35,36]
Loic Le Marchand[37]
Xin Sheng[38]
Elaine A. Ostrander[39]
Milan S. Geybels[40]
Edward Giovannucci[41]
Peter Kraft[42]
Neil Burnet[43]
Gill Barnett[44]
Bettina F. Drake[45]
Robert Szulkin[46,47]
Géraldine Cancel-Tassin[48,49]
Stephen Chanock[50]
Gerald L. Andriole[51]
Laura E. Beane Freeman[52]
Michael Borre[53,54]
Dominika Wokolorczyk[55]
Jan Lubinski[56]
Lovise Maehle[57]
Thérèse Truong[58]
Yves Akoli Koudou[58]
Hui-Yi Lin[60]
Mariana C Stern[61]
Manuel Luedeke[62]

Thomas Schnoeller[63]
Neil E. Fleshner[64]
Antonio Finelli[65]
Shannon K. McDonnell[66]
Daniel J. Schaid[66]
Sarah L. Kerns[67]
Harry Ostrer[68]
Hong-Wei Zhang [69]
Ninghan Feng[70]
Xin Guo[71]
Xueying Mao[72]
Zan Sun[73]
Guoping Ren[74]
Antonio Gómez-Caamaño[75]
Laura Fachal[76,77,78]
Jeannette T. Bensen[79,80]
Elizabeth T.H. Fontham[81]
James Mohler[80,82]
Jack A. Taylor[83,84]
Javier Llorca[85,86]
Gemma Castaño-Vinyals[87,88,89]
Antonio Alcaraz[90]
Martin Eklund[91]
Sara Lindström[92]
Meir Stampfer[93]
Curtis Pettaway[94]
Paula Paulo[95,96]
Andreia Brandão[97,96]
Marta Cardoso[97,96]
Maria P. Silva[97,96]
Pascal Blanchet[98]
Laurent Brureau[98]
Craig C Teerlink[99,100]
Yan Zhang [101]
Bernd Holleczek[102]
Ben Schöttker[101]
Chavdar Slavov[103]
Vanio Mitev[104]
Jeri Kim[105]
Yuan Chun Ding[106]
Linda Steele[106]
Gert De Meerleer[107]
Piet Ost[108]
Agnieszka Michael[109]
Jasmine Lim[110]
Soo-Hwang Teo[111]
Daniel W. Lin[40,112]
Davor Lessel[113]
Tomislav Kulis[114]
Aswin Abraham[115]

Matthew Parliament[115,116]
Steven Joniau[117]
Thomas Van den Broeck[118,117]
Jose Esteban Castelao[119]
Melinda Aldrich[120]
Dana C. Crawford[121]
Jennifer C. Cullen[122,123]
George Petrovics[122,123]
Ron H.N. van Schaik[124]
Guido Jenster[125]

[1]Division of Population Health, Health Services Research and Primary Care, School of Health Sciences, Faculty of Biology, Medicine and Health, University of Manchester, Manchester, UK , M139PT
[2]Department of Urology, Tampere University Hospital, Tampere, Finland
[3]Institute of Biomedicine, Kiinamyllynkatu 10, FI-20014 University of Turku, Finland
[4]Unit of Health Sciences, Faculty of Social Sciences, Tampere University, Tampere, Finland
[5]Centre for Cancer Genetic Epidemiology, Department of Oncology, University of Cambridge, Strangeways Laboratory, Worts Causeway, Cambridge, CB1 8RN,UK
[6]Open Targets, Wellcome Sanger Institute, Hinxton, Saffron Walden, CB10 1SA, UK
[7]University of Technology, Sydney
[8]Cancer Council Queensland, Fortitude Valley, QLD 4006, Australia
[9]Australian Prostate Cancer Research Centre-Qld, Institute of Health and Biomedical Innovation and School of Biomedical Science, Queensland University of Technology, Brisbane, QLD 4059, Australia
[10]Translational Research Institute, Brisbane, Queensland 4102, Australia
[11]Chris O'Brien Lifehouse (COBLH), Camperdown, Sydney, NSW 2010, Australia
[12]Garvan Institute of Medical Research, Sydney NSW 2010, Australia
[13]Australian Prostate Cancer Research Centre-Qld, Institute of Health and Biomedical Innovation and School of Biomedical Sciences, Queensland University of Technology, Brisbane, 4059, Australia
[14]Department of Anatomy and Developmental Biology, Biomedicine Discovery Institute, Monash University, Melbourne, Victoria 3800, Australia
[15]Prostate Cancer Translational Research Program, Cancer Research Division, Peter MacCallum Cancer Centre, Melbourne, VIC 3000, Australia
[16]Dame Roma Mitchell Cancer Research Laboratories, University of Adelaide, Adelaide, South Australia, Australia
[17]Department of Medical Epidemiology and Biostatistics, Karolinska Institute, Stockholm, Sweden
[18]Department of Molecular Medicine and Surgery, Karolinska Institutet, and Department of Urology, Karolinska University Hospital, Solna, 171 76 Stockholm
[19]Department of Clinical Sciences at Danderyds Hospital, Karolinska Institutet, Stockholm, Sweden
[20]Faculty of Health and Medical Sciences, University of Copenhagen, Denmark
[21]Department of Clinical Biochemistry, Herlev and Gentofte Hospital, Copenhagen University Hospital, Herlev, 220 Copenhagen, Denmark
[22]Copenhagen Prostate Cancer Center, Department of Urology, Rigshospitalet, Copenhagen University Hospital, DK-2730 Herlev, Denmark
[23]Copenhagen Prostate Cancer Center, Department of Urology, Rigshospitalet, Copenhagen University Hospital, DK-2730 Herlev, Copenhagen, Denmark
[24]SWOG Statistical Center, Fred Hutchinson Cancer Research Center, Seattle, WA, USA
[25]CHRISTUS Santa Rosa Hospital Medical Center, San Antonio, TX, USA
[26]Precision Medicine, School and Clinical Sciences at Monash Health, Monash University, Clayton, Victoria, 3168.

[27]Cancer Epidemiology Division, Cancer Council Victoria, 615 St Kilda Road, Melbourne, VIC 3004, Australia
[28]Department of Clinical Pathology, The Melbourne Medical School, The University of Melbourne, Melbourne, Victoria, Australia.
[29]Centre for Epidemiology and Biostatistics, Melbourne School of Population and Global Health, The University of Melbourne, Victoria, 3010, Australia
[30]Cancer Epidemiology Division, Cancer Council Victoria, 615 St Kilda Road, Melbourne, Victoria, 3004, Australia
[31]Centre for Epidemiology and Biostatistics, Melbourne School of Population and Global Health, The University of Melbourne, Melbourne, Victoria, Australia.
[32]Precision Medicine, School of Clinical Sciences at Monash Health, Monash University, Clayton, Victoria 3168, Australia
[33]Division of Nutritional Epidemiology, Institute of Environmental Medicine, Karolinska Institutet, SE-171 77 Stockholm, Sweden
[34]Division of Cancer Epidemiology and Genetics, National Cancer Institute, NIH, Bethesda, MD 20892, USA
[35]Department of Population and Quantitative Health Sciences, Case Western Reserve University, Cleveland, OH 44106-7219, USA
[36]Seidman Cancer Center, University Hospitals, Cleveland, OH 44106, USA.
[37]Epidemiology Program, University of Hawaii Cancer Center, Honolulu, HI 96813, USA
[38]Center for Genetic Epidemiology, Department of Preventive Medicine, Keck School of Medicine, University of Southern California/Norris Comprehensive Cancer Center, Los Angeles, CA 90015, USA
[39]National Human Genome Research Institute, National Institutes of Health, 50 South Drive, Rm. 5351, Bethesda, MD 20892, USA
[40]Division of Public Health Sciences, Fred Hutchinson Cancer Research Center, Seattle, Washington, 98109-1024, USA
[41]Department of Epidemiology, Harvard School of Public Health, Boston, MA 02115, USA
[42]Program in Genetic Epidemiology and Statistical Genetics, Department of Epidemiology, Harvard T. H. Chan School of Public Health, Boston 02115, USA
[43]Division of Cancer Sciences, University of Manchester, Manchester Cancer Research Centre, Manchester Academic Health Science Centre, and The Christie NHS Foundation Trust, Manchester, UK
[44]University of Cambridge Department of Oncology, Oncology Centre, Cambridge University Hospitals NHS Foundation Trust, Hills Road, Cambridge, CB2 0QQ, UK
[45]Washington University School of Medicine, 660 S. Euclid Avenue, Campus Box 8242, St. Louis, MO 63110 , USA
[46]Division of Family Medicine, Department of Neurobiology, Care Science and Society, Karolinska Institutet, Huddinge, SE-171 77 Stockholm, Sweden
[47]Scandinavian Development Services, Danderyd, 182 33, Sweden
[48]CeRePP, Tenon Hospital, Paris, France.
[49]Sorbonne Universite, GRC n°5 , AP-HP, Tenon Hospital, 4 rue de la Chine, F-75020 Paris, France
[50]Division of Cancer Epidemiology and Genetics, National Cancer Institute, NIH, Bethesda, MD, 20892, USA
[51]Washington University School of Medicine, St. Louis, MO, USA
[52]Division of Cancer Epidemiology and Genetics, National Cancer Institute, NIH, Bethesda, MD, USA
[53]Department of Urology, Aarhus University Hospital, Palle Juul-Jensen Boulevard 99, 8200 Aarhus N, Denmark
[54]Department of Clinical Medicine, Aarhus University
[55]International Hereditary Cancer Center, Department of Genetics and Pathology, Pomeranian Medical University, 70-115 Szczecin, Poland

[56]International Hereditary Cancer Center, Department of Genetics and Pathology, Pomeranian Medical University, Szczecin, Poland
[57]Department of Medical Genetics, Oslo University Hospital, 0424 Oslo, Norway
[58]Cancer & Environment Group, Center for Research in Epidemiology and Population Health (CESP), INSERM, University Paris-Sud, University Paris-Saclay, 94807 Villejuif CÈdex, France
[60]School of Public Health, Louisiana State University Health Sciences Center, New Orleans, LA 70112, USA
[61]Department of Preventive Medicine, Keck School of Medicine, University of Southern California/Norris Comprehensive Cancer Center, Los Angeles, CA 90015, USA
[62]Humangenetik Tuebingen, Paul-Ehrlich-Str 23, D-72076 Tuebingen
[63]Department of Urology, University Hospital Ulm, Germany
[64]Dept. of Surgical Oncology, Princess Margaret Cancer Centre, Toronto ON M5G 2M9, Canada
[65]Division of Urology, Princess Margaret Cancer Centre, Toronto ON M5G 2M9, Canada
[66]Division of Biomedical Statistics & Informatics, Mayo Clinic, Rochester, MN 55905, USA
[67]Department of Radiation Oncology, University of Rochester Medical Center, Saunders Research Building, Rm 2.202, 265 Crittenden Boulevard, Rochester, NY 14620, USA
[68]Professor of Pathology and Pediatrics, Albert Einstein College of Medicine, 1300 Morris Park Avenue, Ullman 817, Bronx, NY 10461, USA
[69]Second Military Medical University, 800 Xiangyin Rd., Shanghai 200433, P. R. China
[70]Wuxi Second Hospital, Nanjing Medical University, 68 Zhongshan Road, Wuxi, Jiangzhu Province, 214003, China
[71]1 Youyi Road, Department of Urology, The First Affiliated Hospital, Chongqing Medical University, Chongqing, 400016, China
[72]Centre for Molecular Oncology, Barts Cancer Institute, Queen Mary University of London, John Vane Science Centre, Charterhouse Square, London EC1M 6BQ, UK
[73]33 Wenyi Road, The Peoples Hospital of Liaoning Province, The Peoples Hospital of China Medical University, Shenyang, 110016, China
[74]79 Qingchun Road, Department of Pathology, The First Affiliated Hospital, Zhejiang University Medical College, Hangzhou, 310009, China
[75]Department of Radiation Oncology, Complexo Hospitalario Universitario de Santiago, SERGAS, Santiago de Compostela 15706, Spain
[76]Centre for Cancer Genetic Epidemiology, Department of Public Health and Primary Care, University of Cambridge, Strangeways Research Laboratory, Cambridge, CB2 0SR, UK.
[77]Fundación Pública Galega Medicina Xenómica, Santiago De Compostela, 15706, Spain.
[78]Instituto de Investigación Sanitaria de Santiago de Compostela, Santiago De Compostela, 15706, Spain.
[79]Department of Epidemiology, University of North Carolina at Chapel Hill, Chapel Hill, NC, USA
[80]Lineberger Comprehensive Cancer Center, University of North Carolina at Chapel Hill, Chapel Hill, NC, USA
[81]School of Public Health, Louisiana State University Health Sciences Center, New Orleans, LA, USA
[82]Department of Urology, Roswell Park Cancer Institute, Buffalo, NY, USA
[83]Epidemiology Branch, National Institute of Environmental Health Sciences, Research Triangle Park, NC, USA
[84]Epigenetic and Stem Cell Biology Laboratory, National Institute of Environmental Health Sciences, Research Triangle Park, NC
[85]University of Cantabria-IDIVAL, 39005 Santander, Spain
[86] CIBER Epidemiología y Salud Pública (CIBERESP), 28029 Madrid, Spain
[87]ISGlobal, Barcelona, Spain
[88]IMIM (Hospital del Mar Medical Research Institute), Barcelona, Spain
[89]Universitat Pompeu Fabra (UPF), Barcelona, Spain

[90] Department and Laboratory of Urology. Hospital Clínic. Institut d'Investigacions Biomèdiques August Pi i Sunyer (IDIBAPS), Universitat de Barcelona. Spain. C/Villarroel 170; 08036 Barcelona, Spain
[91]Department of Medical Epidemiology and Biostatistics, Karolinska Institute, 171 77 Stockholm, Sweden
[92]Department of Epidemiology, Health Sciences Building, University of Washington
[93]Channing Division of Network Medicine, Department of Medicine, Brigham and Women's Hospital/Harvard Medical School, Boston, MA 02184, USA
[94]The University of Texas M. D. Anderson Cancer Center, 1515 Holcombe Blvd., Houston, TX 77030, USA
[95]Department of Genetics, Portuguese Oncology Institute of Porto (IPO-Porto), 4200-072 Porto, Portugal
[96]Cancer Genetics Group, IPO-Porto Research Center (CI-IPOP), Portuguese Oncology Institute of Porto (IPO-Porto), Porto, Portugal
[97]Department of Genetics, Portuguese Oncology Institute of Porto, Porto, Portugal
[98]CHU CHU de Pointe-à-Pitre, Univ Antilles, Univ Rennes, Inserm, EHESP, Irset (Institut de recherche en santé, environnement et travail) - UMR_S 1085, Pointe-à-Pitre, France
[99]Division of Epidemiology, Department of Internal Medicine, University of Utah School of Medicine
[100]George E. Wahlen Department of Veterans Affairs Medical Center, Salt Lake City, Utah, USA
[101]Division of Clinical Epidemiology and Aging Research, German Cancer Research Center (DKFZ), Heidelberg, Germany
[102]Saarland Cancer Registry, 66119 Saarbrücken, Germany
[103]Department of Urology and Alexandrovska University Hospital, Medical University of Sofia, 1431 Sofia, Bulgaria
[104]Molecular Medicine Center, Department of Medical Chemistry and Biochemistry, Medical University of Sofia, Sofia, 2 Zdrave Str., 1431 Sofia, Bulgaria
[105]The University of Texas M. D. Anderson Cancer Center, Department of Genitourinary Medical Oncology, 1515 Holcombe Blvd., Houston, TX 77030, USA
[106]Department of Population Sciences, Beckman Research Institute of the City of Hope, 1500 East Duarte Road, Duarte, CA 91010, 626-256-HOPE (4673)
[107]Ghent University, B-9000, Gent
[108]Ghent University Hospital, Department of Radiotherapy, De Pintelaan 185, B-9000, Gent, Belgium
[109]The University of Surrey, Guildford, Surrey, GU2 7XH
[110]Department of Surgery, Faculty of Medicine, University of Malaya, 50603 Kuala Lumpur, Malaysia
[111]Cancer Research Malaysia (CRM), Outpatient Centre, Subang Jaya Medical Centre, Subang Jaya, Selangor, Malaysia
[112]Department of Urology, University of Washington, 1959 NE Pacific Street, Box 356510, Seattle, WA 98195, USA
[113]Institute of Human Genetics, University Medical Center Hamburg-Eppendorf, D-20246 Hamburg, Germany
[114]Department of Urology, University Hospital Center Zagreb, University of Zagreb School of Medicine , Zagreb, Croatia
[115]Department of Oncology, Cross Cancer Institute, University of Alberta, 11560 University Avenue, Edmonton, Alberta, Canada T6G 1Z2
[116]Division of Radiation Oncology, Cross Cancer Institute, 11560 University Avenue, Edmonton, Alberta, Canada T6G 1Z2
[117]Department of Urology, University Hospitals Leuven, Herestraat 49, Box 7003 41, BE-3000 Leuven, Belgium
[118]Molecular Endocrinology Laboratory, Department of Cellular and Molecular Medicine, KU BE-3000 Leuven, Belgium
[119]Genetic Oncology Unit, CHUVI Hospital, Complexo Hospitalario Universitario de Vigo, Instituto de Investigación Biomédica Galicia Sur (IISGS), 36204, Vigo (Pontevedra), Spain

[120]Vanderbilt University Medical Center, Department of Thoracic Surgery, 609 Oxford House, 1313 21st Avenue South, Nashville, TN 37232-4682 USA
[121]Case Western Reserve University, Department of Population and Quantitative Health Sciences, Cleveland Institute for Computational Biology, 2103 Cornell Road, Wolstein Research Building, Suite 2527, Cleveland, OH, 44106 USA
[122]Uniformed Services University, 4301 Jones Bridge Rd, Bethesda, MD 20814, USA
[123]Center for Prostate Disease Research, 1530 East Jefferson Street, Rockville, MD 20852, USA
[124]Department of Clinical Chemistry, Erasmus University Medical Center, 3015 CE Rotterdam, The Netherlands
[125]Department of Urology, Erasmus University Medical Center, 3015 CE Rotterdam, The Netherlands

Funding & Acknowledgements

CRUK and PRACTICAL consortium

This work was supported by the Canadian Institutes of Health Research, European Commission's Seventh Framework Programme grant agreement n° 223175 (HEALTH-F2-2009-223175), Cancer Research UK Grants C5047/A7357, C1287/A10118, C1287/A16563, C5047/A3354, C5047/A10692, C16913/A6135, and The National Institute of Health (NIH) Cancer Post-Cancer GWAS initiative grant: No. 1 U19 CA 148537-01 (the GAME-ON initiative).

We would also like to thank the following for funding support: The Institute of Cancer Research and The Everyman Campaign, The Prostate Cancer Research Foundation, Prostate Research Campaign UK (now Prostate Action), The Orchid Cancer Appeal, The National Cancer Research Network UK, The National Cancer Research Institute (NCRI) UK. We are grateful for support of NIHR funding to the NIHR Biomedical Research Centre at The Institute of Cancer Research and The Royal Marsden NHS Foundation Trust.

The Prostate Cancer Program of Cancer Council Victoria also acknowledge grant support from The National Health and Medical Research Council, Australia (126402, 209057, 251533, , 396414, 450104, 504700, 504702, 504715, 623204, 940394, 614296,), VicHealth, Cancer Council Victoria, The Prostate Cancer Foundation of Australia, The Whitten Foundation, PricewaterhouseCoopers, and Tattersall's. EAO, DMK, and EMK acknowledge the Intramural Program of the National Human Genome Research Institute for their support.

Genotyping of the OncoArray was funded by the US National Institutes of Health (NIH) [U19 CA 148537 for ELucidating Loci Involved in Prostate cancer SuscEptibility (ELLIPSE) project and X01HG007492 to the Center for Inherited Disease Research (CIDR) under contract number HHSN268201200008I].

This study would not have been possible without the contributions of the following: Coordination team, bioinformatician and genotyping centers: Genotyping at CCGE, Cambridge: Caroline Baines and Don Conroy

Funding for the iCOGS infrastructure came from: the European Community's Seventh Framework Programme under grant agreement n° 223175 (HEALTH-F2-2009-223175) (COGS), Cancer Research UK (C1287/A10118, C1287/A 10710, C12292/A11174, C1281/A12014, C5047/A8384, C5047/A15007, C5047/A10692, C8197/A16565), the National Institutes of Health (CA128978) and Post-Cancer GWAS initiative (1U19 CA148537, 1U19 CA148065 and 1U19 CA148112 - the GAME-ON initiative), the Department of Defence (W81XWH-10-1-0341), the Canadian Institutes of Health Research (CIHR) for the CIHR Team in Familial Risks of Breast Cancer, Komen Foundation for the Cure, the Breast Cancer Research Foundation, and the Ovarian Cancer Research Fund.

This study would not have been possible without the contributions of the following: Per Hall (COGS); Douglas F. Easton, Paul Pharoah, Kyriaki Michailidou, Manjeet K. Bolla, Qin Wang (BCAC), Andrew Berchuck (OCAC), Rosalind A. Eeles, Douglas F. Easton, Ali Amin Al Olama, Zsofia Kote-Jarai, Sara Benlloch (PRACTICAL), Georgia Chenevix-Trench, Antonis Antoniou, Lesley McGuffog, Fergus Couch and Ken Offit (CIMBA), Joe Dennis, Alison M. Dunning, Andrew Lee, and Ed Dicks, Craig Luccarini and the staff of the Centre for Genetic Epidemiology Laboratory, Javier Benitez, Anna Gonzalez-Neira and the staff of the CNIO genotyping unit, Jacques Simard and Daniel C. Tessier, Francois Bacot, Daniel Vincent, Sylvie LaBoissière and Frederic Robidoux and the staff of the McGill University and Génome Québec Innovation Centre, Stig E. Bojesen, Sune F. Nielsen, Borge G.

Nordestgaard, and the staff of the Copenhagen DNA laboratory, and Julie M. Cunningham, Sharon A. Windebank, Christopher A. Hilker, Jeffrey Meyer and the staff of Mayo Clinic Genotyping Core Facility

Additional funding and acknowledgments from studies in PRACTICAL:

Information of the PRACTICAL consortium can be found at http://practical.icr.ac.uk/

Aarhus
This study was supported by Innovation Fund Denmark, the Danish Cancer Society and The Velux Foundation (Veluxfonden). The Danish Cancer Biobank (DCB) is acknowledged for biological material.

AHS
This work was supported by the Intramural Research Program of the NIH, National Cancer Institute, Division of Cancer Epidemiology and Genetics (Z01CP010119).

ATBC
The ATBC Study is supported by the Intramural Research Program of the U.S. National Cancer Institute, National Institutes of Health, and by U.S. Public Health Service contract HHSN261201500005C from the National Cancer Institute, Department of Health and Human Services.

BioVU
The dataset(s) used for the analyses described were obtained from Vanderbilt University Medical Center's BioVU which is supported by institutional funding and by the National Center for Research Resources, Grant UL1 RR024975-01 (which is now at the National Center for Advancing Translational Sciences, Grant 2 UL1 TR000445-06).

Canary PASS
PASS was supported by Canary Foundation and the National Cancer Institute's Early Detection Research Network (U01 CA086402)

CAPS & STHM1
The Department of Medical Epidemiology and Biostatistics, Karolinska Institute, Stockholm, Sweden was supported by the Cancer Risk Prediction Center (CRisP; www.crispcenter.org), a Linneus Centre (Contract ID 70867902) financed by the Swedish Research Council, Swedish Research Council (grant no K2010-70X-20430-04-3), the Swedish Cancer Foundation (grant no 09-0677), the Hedlund Foundation, the Soederberg Foundation, the Enqvist Foundation, ALF funds from the Stockholm County Council. Stiftelsen Johanna Hagstrand och Sigfrid Linner's Minne, Karlsson's Fund for urological and surgical research. We thank and acknowledge all of the participants in the Stockholm-1 study. We thank Carin Cavalli-Bjoerkman and Ami Roennberg Karlsson for their dedicated work in the collection of data. Michael Broms is acknowledged for his skilful work with the databases. KI Biobank is acknowledged for handling the samples and for DNA extraction. Hans Wallinder at Aleris Medilab and Sven Gustafsson at Karolinska University Laboratory are thanked for their good cooperation in providing historical laboratory results.

CCI
This work was awarded by Prostate Cancer Canada and is proudly funded by the Movember Foundation - Grant # D2013-36. The CCI group would like to thank David Murray, Razmik Mirzayans, and April Scott for their contribution to this work.

CHIPGECS

Orchid, Wuxi Second Hospital Research Funds, National Natural Science foundation of China for funding support to H Zhang (Grant No: 30671793 and 81072377), N Feng (Grant No: 81272831), SC Zhao (Grant No: 81072092 and 81328017), Liaoning Natural Science Foundation 2017, China, Item Number: 20170540536 to Zan Sun and Yong-Jie Lu. This work was conducted on behalf of the CHIPGECS Consortia. We acknowledge the contribution of doctors, nurses and postgraduate research students at the CHIPGENCS sample collecting centers.

COH

SLN is partially supported by the Morris and Horowitz Families Endowed Professorship

CONOR

CONOR was supported by grants from the Nordic Cancer Union, the Swedish Cancer Society (2012/823) and the Swedish Research Council (2014/2269). The authors wish to acknowledge the services of CONOR, the contributing research centres delivering data to CONOR, and all the study participants.

COSM

COSM is funded by The Swedish Research Council (grant for the Swedish Infrastructure for Medical Population-based Life-course Environmental Research – SIMPLER), the Swedish Cancer Foundation.

CPCS1 / CPCS2

Department of Clinical Biochemistry, Herlev and Gentofte Hospital, Copenhagen University Hospital, Herlev Ringvej 75, DK-2730 Herlev, Denmark We thank participants and staff of the Copenhagen General Population Study for their important contributions.

CPDR

Uniformed Services University for the Health Sciences HU0001-10-2-0002 (PI: David G. McLeod, MD)

EPIC

The coordination of EPIC was financially supported by the European Commission (DG-SANCO) and the International Agency for Research on Cancer. The national cohorts (that recruited male participants) are supported by Danish Cancer Society (Denmark); German Cancer Aid, German Cancer Research Center (DKFZ), Federal Ministry of Education and Research (BMBF), Deutsche Krebshilfe, Deutsches Krebsforschungszentrum and Federal Ministry of Education and Research (Germany); the Hellenic Health Foundation (Greece); Associazione Italiana per la Ricerca sul Cancro-AIRC-Italy and National Research Council (Italy); Dutch Ministry of Public Health, Welfare and Sports (VWS), Netherlands Cancer Registry (NKR), LK Research Funds, Dutch Prevention Funds, Dutch ZON (Zorg Onderzoek Nederland), World Cancer Research Fund (WCRF), Statistics Netherlands (The Netherlands); Health Research Fund (FIS), PI13/00061 to Granada; , PI13/01162 to EPIC-Murcia), Regional Governments of Andalucía, Asturias, Basque Country, Murcia and Navarra, ISCIII RETIC (RD06/0020) (Spain); Swedish Cancer Society, Swedish Research Council and County Councils of Skåne and Västerbotten (Sweden); Cancer Research UK (14136 to EPIC-Norfolk; C570/A16491 and C8221/A19170 to EPIC-Oxford), Medical Research Council (1000143 to EPIC-Norfolk, MR/M012190/1 to EPIC-Oxford) (United Kingdom). For information on how to submit an application for gaining access to EPIC data and/or biospecimens, please follow the instructions at http://epic.iarc.fr/access/index.php

EPICAP

The EPICAP study was supported by grants from Ligue Nationale Contre le Cancer; Institut National du Cancer (INCa); Fondation ARC; Fondation de France; Agence Nationale de sécurité sanitaire de l'alimentation, de l'environnement et du travail (ANSES); Ligue départementale du Val de Marne.

The EPICAP study group would like to thank all urologists, Antoinette Anger and Hasina Randrianasolo (study monitors), Anne-Laure Astolfi, Coline Bernard, Oriane Noyer, Marie-Hélène De Campo, Sandrine Margaroline, Louise N'Diaye, Sabine Perrier-Bonnet (Clinical Research nurses)

ERSPC

This study was supported by the DutchCancerSociety(KWF94-869,98-1657,2002-277,2006-3518, 2010-4800); The Netherlands Organisation for HealthResearch and Development (ZonMW-002822820,22000106,50-50110-98-311, 62300035), The Dutch Cancer Research Foundation(SWOP), and an unconditional grant from Beckman-Coulter-HybritechInc.

ESTHER

The ESTHER study was supported by a grant from the Baden Württemberg Ministry of Science, Research and Arts. The ESTHER group would like to thank Hartwig Ziegler, Sonja Wolf, Volker Hermann, Heiko Müller, Karina Dieffenbach, Katja Butterbach for valuable contributions to the study.

FHCRC

The FHCRC studies were supported by grants R01-CA056678, R01-CA082664, R01-CA092579, and K05-CA175147 from the US National Cancer Institute, National Institutes of Health, with additional support from the Fred Hutchinson Cancer Research Center (P30-CA015704). We thank all the men who participated in these studies.

Gene-PARE

The Gene-PARE study was supported by grants 1R01CA134444 from the U.S. National Institutes of Health, PC074201 and W81XWH-15-1-0680 from the Prostate Cancer Research Program of the Department of Defense and RSGT-05-200-01-CCE from the American Cancer Society. S.L.K. is supported by 1K07CA187546 from the U.S. National Cancer Institute.

HPFS

The Health Professionals Follow-up Study was supported by grants UM1CA167552, CA133891, CA141298, and P01CA055075. We are grateful to the participants and staff of the Physicians' Health Study and Health Professionals Follow-Up Study for their valuable contributions, as well as the following state cancer registries for their help: AL, AZ, AR, CA, CO, CT, DE, FL, GA, ID, IL, IN, IA, KY, LA, ME, MD, MA, MI, NE, NH, NJ, NY, NC, ND, OH, OK, OR, PA, RI, SC, TN, TX, VA, WA, WY.

IMPACT

The IMPACT study was funded by The Ronald and Rita McAulay Foundation, CR-UK Project grant (C5047/A1232), Cancer Australia, AICR Netherlands A10-0227, Cancer Australia and Cancer Council Tasmania, NIHR, EU Framework 6, Cancer Councils of Victorial and South Australia, Philanthropic donation to Northshore University Health System. We acknowledge support from the National Institute for Health Research (NIHR) to the Biomedical Research Centre at The Institute of Cancer Research and Royal Marsden Foundation NHS Trust. We acknowledge the IMPACT study steering committee, collaborating centres and participants.

IPO-Porto

The IPO-Porto study was funded by Fundação para a Ciência e a Tecnologia (FCT; UID/DTP/00776/2013 and PTDC/DTP-PIC/1308/2014) and FEDER (POCI-01-0145-FEDER-028245).

MC (SFRH/BD/116557/2016) and MPS (SFRH/BD/132441/2017) are research fellows from FCT. We would like to express our gratitude to all patients and families who have participated in this study.

KARUPROSTATE
The Karuprostate study was supported by the the Frech National Health Directorate, the Association pour la Recherche sur le Cancer, la Ligue Nationale contre le Cancer, the French Agency for Environmental and Occupational Health Safety (ANSES) and by the Association pour la Recherche sur les Tumeurs de la Prostate. We would like to thank Séverine Ferdinand for valuable contributions to the study.
KULEUVEN. F.C. and S.J. are holders of grants from FWO Vlaanderen (G.0684.12N and G.0830.13N), the Belgian federal government (National Cancer Plan KPC_29_023), and a Concerted Research Action of the KU Leuven (GOA/15/017). TVDB is holder of a doctoral fellowship of the FWO.

LAAPC
This study was funded by grant R01CA84979 (to S.A. Ingles) from the National Cancer Institute, NIH.

Malaysia
The study was funded by the University Malaya High Impact Research Grant (HIR/MOHE/MED/35 to A.R). We thank all associates in the Urology Unit, University of Malaya, Cancer Research Malaysia (CRM) and the Malaysian Men's Health Initiative (MMHI).

MAYO
The Mayo group was supported by the US National Cancer Institute (R01CA72818)

MCC-Spain
The study was partially funded by the ""Accion Transversal del Cancer"", approved on the Spanish Ministry Council on the 11th October 2007, by the Instituto de Salud Carlos III-FEDER (PI08/1770, PI09/00773-Cantabria, PI11/01889-FEDER, PI12/00265, PI12/01270, PI12/00715, PI15/00069), by the Fundación Marqués de Valdecilla (API 10/09), by the Spanish Association Against Cancer (AECC) Scientific Foundation and by the Catalan Government DURSI grant 2009SGR1489. Samples: Biological samples were stored at the Parc de Salut MAR Biobank (MARBiobanc; Barcelona) which is supported by Instituto de Salud Carlos III FEDER (RD09/0076/00036). Also sample collection was supported by the Xarxa de Bancs de Tumors de Catalunya sponsored by Pla Director d'Oncologia de Catalunya (XBTC). ISGlobal is a member of the CERCA Programme, Generalitat de Catalunya. We acknowledge the contribution from Esther Gracia-Lavedan in preparing the data. We thank all the subjects who participated in the study and all MCC-Spain collaborators.

MCCS
Melbourne Collaborative Cohort Study (MCCS) cohort recruitment was funded by VicHealth and Cancer Council Victoria. The MCCS was further augmented by Australian National Health and Medical Research Council grants 209057, 396414 and 1074383 and by infrastructure provided by Cancer Council Victoria. Cases and their vital status were ascertained through the Victorian Cancer Registry and the Australian Institute of Health and Welfare, including the National Death Index and the Australian Cancer Database.

MD Anderson
Prostate Cancer Case-Control Studies at MD Anderson (MDA) supported by grants CA68578, ES007784, DAMD W81XWH-07-1-0645 and CA140388.

MEC
The MEC was supported by NIH grants CA063464, CA054281, CA098758, and CA164973.

MOFFITT
The Moffitt group was supported by the US National Cancer Institute (R01CA128813, PI: J.Y. Park).

NMHS
Funding for the Nashville Men's Health Study (NMHS) was provided by the National Institutes of Health Grant numbers: RO1CA121060

PCaP
The North Carolina - Louisiana Prostate Cancer Project (PCaP) and the Health Care Access and Prostate Cancer Treatment in North Carolina (HCaP-NC) study are carried out as collaborative studies supported by the Department of Defense contract DAMD 17-03-2-0052 and the American Cancer Society award RSGT-08-008-01-CPHPS, respectively. The authors thank the staff, advisory committees and research subjects participating in the PCaP study for their important contributions. We would like to acknowledge the UNC BioSpecimen Facility and the LSUHSC Pathology Lab for our DNA extractions, blood processing, storage, and sample disbursement (https://genome.unc.edu/bsp). The authors thank the staff, advisory committees and research subjects participating in the PCaP and HCaP-NC studies for their important contributions.

PCMUS
The PCMUS study was supported by the Bulgarian National Science Fund, Ministry of Education and Science (contract DOO-119/2009; DUNK01/2-2009; DFNI-B01/28/2012) with additional support from the Science Fund of Medical University - Sofia (contract 51/2009; 8I/2009; 28/2010;).

PHS
The Physicians' Health Study was supported by grants CA34944, CA40360, CA097193, HL26490 and HL34595. We are grateful to the participants and staff of the Physicians' Health Study and Health Professionals Follow-Up Study for their valuable contributions, as well as the following state cancer registries for their help: AL, AZ, AR, CA, CO, CT, DE, FL, GA, ID, IL, IN, IA, KY, LA, ME, MD, MA, MI, NE, NH, NJ, NY, NC, ND, OH, OK, OR, PA, RI, SC, TN, TX, VA, WA, WY.

PLCO
This PLCO study was supported by the Intramural Research Program of the Division of Cancer Epidemiology and Genetics, National Cancer Institute, NIH. The authors thank Drs. Christine Berg and Philip Prorok, Division of Cancer Prevention at the National Cancer Institute, the screening center investigators and staff of the PLCO Cancer Screening Trial for their contributions to the PLCO Cancer Screening Trial. We thank Mr. Thomas Riley, Mr. Craig Williams, Mr. Matthew Moore, and Ms. Shannon Merkle at Information Management Services, Inc., for their management of the data and Ms. Barbara O'Brien and staff at Westat, Inc. for their contributions to the PLCO Cancer Screening Trial. We also thank the PLCO study participants for their contributions to making this study possible.

PRAGGA
PRAGGA was supported by Programa Grupos Emergentes, Cancer Genetics Unit, CHUVI Vigo Hospital, Instituto de Salud Carlos III, Spain. PRAGGA wishes to thank Victor Muñoz Garzón, Manuel Enguix Castelo, Sara Miranda Ponte, Carmen M Redondo, Manuel Calaza, Francisco Gude Sampedro, Joaquín González-Carreró and the staff of the Department of Pathology and Biobank of University Hospital Complex of Vigo, Instituto de Investigación Sanitaria Galicia Sur (IISGS), SERGAS, Vigo,

Spain; Máximo Fraga, José Antúnez and the Biobank of University Hospital Complex of Santiago, Santiago de Compostela, Spain; and Maria Torres, Angel Carracedo and the Galician Foundation of Genomic Medicine.

PROCAP

PROCAP was supported by the Swedish Cancer Foundation (08-708, 09-0677). We thank and acknowledge all of the participants in the PROCAP study. We thank Carin Cavalli-Björkman and Ami Rönnberg Karlsson for their dedicated work in the collection of data. Michael Broms is acknowledged for his skilful work with the databases. KI Biobank is acknowledged for handling the samples and for DNA extraction. We acknowledge The NPCR steering group: Pär Stattin (chair), Anders Widmark, Stefan Karlsson, Magnus Törnblom, Jan Adolfsson, Anna Bill-Axelson, Ove Andrén, David Robinson, Bill Pettersson, Jonas Hugosson, Jan-Erik Damber, Ola Bratt, Göran Ahlgren, Lars Egevad, and Roy Ehrnström.

PROFILE

We would like to acknowledge the support of the Ronald and Rita McAulay Foundation and Cancer Research UK. We also acknowledge support from the National Institute for Health Research (NIHR) to the Biomedical Research Centre at The Institute of Cancer Research and Royal Marsden Foundation NHS Trust . We acknowledge the Profile study steering committee and participants.

PROGReSS

This research was supported by Spanish Instituto de Salud Carlos III (ISCIII) funding, an initiative of the Spanish Ministry of Economy and Innovation partially supported by European Regional Development FEDER Funds (INT15/00070, INT16/00154, INT17/00133; PI19/01424; PI16/00046; PI13/02030; PI10/00164), and through the Autonomous Government of Galicia (Consolidation and structuring program: IN607B) given to A.Vega. We would like to thank the patients for their contribution to the study

ProMPT & ProtecT

ProtecT funding: NIHR

ProMPT Funding*:* CRUK, NIHR, MRC, Cambridge Biomedical Research Centre

ProtecT would like to acknowledge the support of The University of Cambridge, Cancer Research UK. Cancer Research UK grants [C8197/A10123] and [C8197/A10865] supported the genotyping team. We would also like to acknowledge the support of the National Institute for Health Research which funds the Cambridge Bio-medical Research Centre, Cambridge, UK. We would also like to acknowledge the support of the National Cancer Research Prostate Cancer: Mechanisms of Progression and Treatment (PROMPT) collaborative (grant code G0500966/75466) which has funded tissue and urine collections in Cambridge. We are grateful to staff at the Welcome Trust Clinical Research Facility, Addenbrooke's Clinical Research Centre, Cambridge, UK for their help in conducting the ProtecT study. We also acknowledge the support of the NIHR Cambridge Biomedical Research Centre, the NIR HTA (ProtecT grant) and the NCRI / MRC (ProMPT grant) for help with the bio-repository. The UK Department of Health funded the ProtecT study through the NIHR Health Technology Assessment Programme (projects 96/20/06, 96/20/99). The ProtecT trial and its linked ProMPT and CAP (The Cluster Randomized Trial of PSA Testing for Prostate Cancer) studies are supported by Department of Health, England; Cancer Research UK grant number C522/A8649, C11043/A4286, C18281/A8145, C18281/A11326, and C18281/A15064, Medical Research Council of England grant number G0500966, ID 75466 and The NCRI, UK. The epidemiological data for ProtecT were generated though funding from the Southwest National Health Service Research and Development. DNA extraction in ProtecT was supported by USA Dept of Defense award W81XWH-

04-1-0280, Yorkshire Cancer Research and Cancer Research UK. The authors would like to acknowledge the contribution of all members of the ProtecT study research group. Richard Martin was supported by a Cancer Research UK Programme Grant (C18281/A19169) and the National Institute for Health Research Bristol Nutrition Biomedical Research Centre based at University Hospitals Bristol NHS Foundation Trust and the University of Bristol.

The views and opinions expressed therein are those of the authors and do not necessarily reflect those of the Department of Health of England. The bio-repository from ProtecT is supported by the NCRI (ProMPT) Prostate Cancer Collaborative and the Cambridge BMRC grant from NIHR.

We acknowledge support from the National Cancer Research Institute (National Institute of Health Research (NIHR) Collaborative Study: "Prostate Cancer: Mechanisms of Progression and Treatment (PROMPT)" (grant G0500966/75466).

We thank the National Institute for Health Research, Hutchison Whampoa Limited, the Human Research Tissue Bank (Addenbrooke's Hospital), and Cancer Research UK. The authors would like to thank those men with prostate cancer and the subjects who have donated their time and their samples to the Cambridge Biorepository, which were used in this research. We also would like to acknowledge to support of the research staff in S4 who so carefully curated the samples and the follow-up data (Jo Burge, Marie Corcoran, Anne George, and Sara Stearn).

PROtEuS

PROtEuS was supported financially through grants from the Canadian Cancer Society [13149, 19500, 19864, 19865] and the Cancer Research Society, in partnership with the Ministère de l'enseignement supérieur, de la recherche, de la science et de la technologie du Québec, and the Fonds de la recherche du Québec - Santé. PROtEuS would like to thank its collaborators and research personnel, and the urologists involved in subjects recruitment. We also wish to acknowledge the special contribution made by Ann Hsing and Anand Chokkalingam to the conception of the genetic component of PROtEuS.

QLD

The QLD research is supported by The National Health and Medical Research Council (NHMRC) Australia Project Grants [390130, 1009458] and NHMRC Career Development Fellowship, Cancer Australia PdCCRS and Cancer Council Queensland funding to J Batra. The QLD team would like to acknowledge and sincerely thank the urologists, pathologists, data managers and patient participants who have generously and altruistically supported the QLD cohort.

RAPPER

RAPPER is supported by Cancer Research UK [C1094/A11728; C1094/A18504] and Experimental Cancer Medicine Centre funding [C1467/A7286], and the NIHR Manchester Biomedical Research Centre. The RAPPER group thank Dr. Holly Summersgill for project management.

SABOR

The SABOR research is supported by NIH/NCI Early Detection Research Network, grant U01 CA0866402-18. Also supported by the Cancer Center Support Grant to the Mays Cancer Center from the National Cancer Institute (US) P30 CA054174

SCCS

SCCS is funded by NIH grant R01 CA092447, and SCCS sample preparation was conducted at the Epidemiology Biospecimen Core Lab that is supported in part by the Vanderbilt-Ingram Cancer Center (P30 CA68485). Data on SCCS cancer cases used in this publication were provided by the Alabama

Statewide Cancer Registry; Kentucky Cancer Registry, Lexington, KY; Tennessee Department of Health, Office of Cancer Surveillance; Florida Cancer Data System; North Carolina Central Cancer Registry, North Carolina Division of Public Health; Georgia Comprehensive Cancer Registry; Louisiana Tumor Registry; Mississippi Cancer Registry; South Carolina Central Cancer Registry; Virginia Department of Health, Virginia Cancer Registry; Arkansas Department of Health, Cancer Registry, 4815 W. Markham, Little Rock, AR 72205. The Arkansas Central Cancer Registry is fully funded by a grant from National Program of Cancer Registries, Centers for Disease Control and Prevention (CDC). Data on SCCS cancer cases from Mississippi were collected by the Mississippi Cancer Registry which participates in the National Program of Cancer Registries (NPCR) of the Centers for Disease Control and Prevention (CDC). The contents of this publication are solely the responsibility of the authors and do not necessarily represent the official views of the CDC or the Mississippi Cancer Registry.

SCPCS
SCPCS is funded by CDC grant S1135-19/19, and SCPCS sample preparation was conducted at the Epidemiology Biospecimen Core Lab that is supported in part by the Vanderbilt-Ingram Cancer Center (P30 CA68485).

SEARCH
SEARCH is funded by a programme grant from Cancer Research UK [C490/A10124] and supported by the UK National Institute for Health Research Biomedical Research Centre at the University of Cambridge. The University of Cambridge has received salary support in respect of PP from the NHS in the East of England through the Clinical Academic Reserve.

SFPCS
SFPCS was funded by California Cancer Research Fund grant 99-00527V-10182

SNP_Prostate_Ghent
The study was supported by the National Cancer Plan, financed by the Federal Office of Health and Social Affairs, Belgium.

SPAG
Manchester Cancer Research Centre and MRC – Medical Research Council for PhD studentship work, MUG - Male Uprising in Guernsey, Hope for Guernsey and Wessex Medical Research for research support

STHM2
STHM2 was supported by grants from The Strategic Research Programme on Cancer (StratCan), Karolinska Institutet; the Linné Centre for Breast and Prostate Cancer (CRISP, number 70867901), Karolinska Institutet; The Swedish Research Council (number K2010-70X-20430-04-3) and The Swedish Cancer Society (numbers 11-0287 and 11-0624); Stiftelsen Johanna Hagstrand och Sigfrid Linnérs minne; Swedish Council for Working Life and Social Research (FAS), number 2012-0073

The authors acknowledge the Karolinska University Laboratory, Aleris Medilab, Unilabs and the Regional Prostate Cancer Registry for performing analyses and help to retrieve data. Carin Cavalli–Björkman and Britt-Marie Hune for their enthusiastic work as research nurses. Astrid Björklund for skilful data management. We wish to thank the BBMRI.se biobank facility at Karolinska Institutet for biobank services.

SWOG-PCPT and SWOG-SELECT

PCPT and SELECT are funded by Public Health Service grants U10CA37429 and 5UM1CA182883 from the National Cancer Institute. The authors thank the site investigators and staff and, most importantly, the participants from PCPT who donated their time to this trial.

TAMPERE
The Tampere (Finland) study was supported by the Academy of Finland (251074), The Finnish Cancer Organisations, Sigrid Juselius Foundation, and the Competitive Research Funding of the Tampere University Hospital (X51003). The PSA screening samples were collected by the Finnish part of ERSPC (European Study of Screening for Prostate Cancer). TAMPERE would like to thank Riina Liikanen, Liisa Maeaettaenen and Kirsi Talala for their work on samples and databases.

Toronto
Prostate Cancer Canada Movember Discovery Grant (D2013-17) to RJH; Canadian Cancer Society Research Institute Career Development Award in Cancer Prevention (2013-702108) to RJH

UKGPCS
UKGPCS would also like to thank the following for funding support: The Institute of Cancer Research and The Everyman Campaign, The Prostate Cancer Research Foundation, Prostate Research Campaign UK (now Prostate Action), The Orchid Cancer Appeal, The National Cancer Research Network UK, The National Cancer Research Institute (NCRI) UK. We are grateful for support of NIHR funding to the NIHR Biomedical Research Centre at The Institute of Cancer Research and The Royal Marsden NHS Foundation Trust. UKGPCS should also like to acknowledge the NCRN nurses, data managers and Consultants for their work in the UKGPCS study. UKGPCS would like to thank all urologists and other persons involved in the planning, coordination, and data collection of the study. KM and AL were in part supported from the NIHR Manchester Biomedical Research Centre

ULM
The Ulm group received funds from the German Cancer Aid (Deutsche Krebshilfe).

UTAH
UTAH thank The Keith and Susan Warshaw Fund, C. S. Watkins Urologic Cancer Fund and The Tennity Family Fund supported the Utah study. The project was supported by Award Number P30CA042014 from the National Cancer Institute

WUGS
WUGS would like to thank the following for funding support: The Anthony DeNovi Fund, the Donald C. McGraw Foundation, and the St. Louis Men's Group Against Cancer.

References from Supplemental Material

1. Schumacher FR, Al Olama AA, Berndt SI, et al. Association analyses of more than 140,000 men identify 63 new prostate cancer susceptibility loci. *Nat Genet*. 2018;50(7):928-936. doi:10.1038/s41588-018-0142-8
2. Amos CI, Dennis J, Wang Z, et al. The OncoArray consortium: A network for understanding the genetic architecture of common cancers. *Cancer Epidemiol Biomarkers Prev*. 2017;26(1):126-135. doi:10.1158/1055-9965.EPI-16-0106
3. Li Y, Byun J, Cai G, et al. FastPop: A rapid principal component derived method to infer intercontinental ancestry using genetic data. *BMC Bioinformatics*. 2016;17(1). doi:10.1186/s12859-016-0965-1
4. Karunamuni RA, Huynh-Le M-P, Fan CC, et al. The effect of sample size on polygenic hazard models for prostate cancer. *Eur J Hum Genet*. June 2020. doi:10.1038/s41431-020-0664-2
5. Raj A, Stephens M, Pritchard JK. FastSTRUCTURE: Variational inference of population structure in large SNP data sets. *Genetics*. 2014;197(2):573-589. doi:10.1534/genetics.114.164350
6. Seibert TM, Fan CC, Wang Y, et al. Polygenic hazard score to guide screening for aggressive prostate cancer: Development and validation in large scale cohorts. *BMJ*. 2018;360:1-7. doi:10.1136/bmj.j5757
7. Noone AM, Howlader N, Krapcho M, Miller D, Brest A, Yu M, Ruhl J, Tatalovich Z, Mariotto A, Lewis DR, Chen HS, Feuer EJ CK (eds). SEER Cancer Statistics Review, 1975-2015. National Cancer Institute, Bethesda, MD. https://seer.cancer.gov/csr/1975_2015/. Accessed March 5, 2019.
8. Engholm G, Ferlay J, Christensen N, et al. NORDCAN - A Nordic tool for cancer information, planning, quality control and research. *Acta Oncol (Madr)*. 2010;49(5):725-736. doi:10.3109/02841861003782017
9. Engholm G, Ferlay J, Christensen N, et al. Cancer Incidence, Mortality, Prevalence and Survival in the Nordic Countries, Version 8.1 (28.06.2018). Association of the Nordic Cancer Registries. Danish Cancer Society. http://www.ancr.nu. Accessed September 18, 2018.
10. Statistics Sweden. Population statistics. https://www.scb.se/en/finding-statistics/statistics-by-subject-area/population/population-composition/population-statistics/. Accessed March 5, 2019.
11. Socialstyrelsen - The National Board of Health and Welfare. Statistics on Cancer Incidence 2016. https://www.socialstyrelsen.se/globalassets/sharepoint-dokument/artikelkatalog/statistik/2017-12-31.pdf. Accessed March 5, 2019.
12. Cancer Research UK. Prostate cancer incidence statistics. https://www.cancerresearchuk.org/health-professional/cancer-statistics/statistics-by-cancer-type/prostate-cancer/incidence. Accessed August 15, 2018.
13. Australian Institute of Health and Welfare. *2017 Australian Cancer Incidence and Mortality (ACIM) Books: Prostate Cancer*. Canberra: AIHW.: http://www.aihw.gov.au/acim-books
14. Therneau TM, Li H. Computing the Cox Model for Case Cohort Designs. *Lifetime Data Anal*. 1999;5(2):99-112. doi:10.1023/A:1009691327335
15. Therneau TM, Grambsch PM. *Modeling Survival Data: Extending the Cox Model*. New York: Springer; 2000.
16. R Core Team. R: A language and environment for statistical computing. In: *Vienna, Austria: R Foundation for Statistical Computing*. ; 2015.
17. Rebbeck TR. Prostate Cancer Genetics: Variation by Race, Ethnicity, and Geography. *Semin Radiat Oncol*. 2017;27(1):3-10. doi:10.1016/j.semradonc.2016.08.002
18. Taitt HE. Global Trends and Prostate Cancer: A Review of Incidence, Detection, and Mortality as Influenced by Race, Ethnicity, and Geographic Location. *Am J Mens Health*. 2018;12(6):1807-1823. doi:10.1177/1557988318798279

19. Klein JP, Houwelingen HC, Ibrahim JG ST, ed. *Handbook of Survival Analysis*. London: Chapman and Hall/CRC; 2013.
