# Supplementary material for: Polygenic hazard score is associated with prostate cancer in multi-ethnic populations
Source: Nat Commun. 2021 Feb 23;12:1236. doi: 10.1038/s41467-021-21287-0 (PMC7902617; doi:10.1038/s41467-021-21287-0)
Supplement: Supplementary file 3 — Description of Additional Supplementary Files [file 41467_2021_21287_MOESM3_ESM.pdf]

Description of Additional Supplementary Files

File Name: Supplementary Software 1
Description: Code used in analyses
